# Analysis of the association between serum antiaging humoral factor klotho and cardiovascular disease potential risk factor apolipoprotein B in general population

**DOI:** 10.1097/MD.0000000000034056

**Published:** 2023-06-23

**Authors:** Zhiyi Chen, Tao Tao, Guixiao Huang, Xin Tong, Qinhe Li, Guanyu Su

**Affiliations:** a Department of Urology, The Affiliated Luohu Hospital of Shenzhen University, Shenzhen University School of Medicine, Shenzhen, China; b Shenzhen Following Precision Medical Research Institute, Luohu Hospital Group, Shenzhen, China; c Shantou University Medical College, Shantou, China.

**Keywords:** ApoB, CVD, Klotho, NHANES

## Abstract

Cardiovascular disease (CVD) is a prevalent health issue, and various risk factors contribute to its development, including blood lipids, blood pressure, diabetes, smoking, and alcohol consumption. Apolipoprotein B (ApoB) is related to CVD. ApoB is present on the surface of low-density lipoprotein (LDL), and its cellular recognition and LDL uptake are mainly achieved through recognition. It plays a crucial role in the diagnosis and treatment of CVD. This study aims to investigate the relationship between Klotho and ApoB in the general population of the United States as the correlation between serum Klotho and apoB is currently unknown. These findings could potentially guide the development of future treatments for CVD. This study utilized data from the National Health and Nutrition Examination Survey (NHANES) collected between 2007 and 2016. A linear regression model and smooth curve fitting were conducted to analyze the relationship between serum Klotho and apoB. The results indicate a negative correlation between serum Klotho concentration and apoB concentration (β = −71.7; 95% confidence interval [CI]: −120.8, −22.6; *P* = .005). After adjusting for confounding variables, the negative correlation between apoB concentration and serum Klotho concentration became more significant (β = −91.8; 95% CI: −151.3, −32.2; *P* = .004). When apoB concentration was converted from a continuous variable to a categorical variable (tertiles: T1 <0.8 g/L; T2: ≥0.8 g/L to <1.0 g/L; T3: ≥1.0 g/L), the serum klotho level of participants in the highest tertile (≥1.0 g/L) was −44.8 pg/mL (95% CI: −86.3, −3.2; *P* = .040) lower than that in the lowest tertile (<0.8 g/L). The smooth curve fitting diagram revealed differences in the relationship between serum Klotho concentration and apoB among individuals with different CVD risk factors. This study demonstrates a significant negative correlation between serum Klotho concentration and apoB concentration, even after controlling for confounding factors. The findings suggest that serum Klotho and apoB may be involved in the development of CVD, and targeting these factors could be a potential approach for CVD prevention and treatment.

## 1. Introduction

Klotho is a kidney protective factor that has been extensively studied. The human Klotho gene, located on chromosome 13, encodes Ι Type single transmembrane glycoprotein membrane binding Klotho. Its relative molecular weight is 130 kDa, and it is composed of 1012 amino acids.^[1]^ Membrane-bound Klotho passes through α and β Cut to form a truncated α-Klotho protein, also known as soluble α-Klotho protein.^[2]^ The Klotho protein discussed in this paper is α-Klotho, which is widely expressed in various human tissues, with the kidney having the highest level of Klotho gene and protein expression.^[3]^ Specifically, Klotho is mainly expressed in the distal convoluted tubules of the kidney and only a small amount in the proximal convoluted tubules.^[4]^ Identified in 1997 as an anti-aging gene, Klotho is also closely related to kidney function and acts as a renal protective agent.^[5–8]^ Additionally, Klotho can serve as a marker of chronic kidney disease (CKD), as the level of Klotho in the kidney of CKD patients is significantly lower than that in a regular control group.^[9]^ In experiments with rats with diabetic nephropathy, azithromycin nephropathy, or spontaneous hypertension, supplementation of Klotho was found to reduce podocyte and glomerular hypertrophy, indicating its protective role in the kidney.^[10–12]^ The relationship between serum Klotho and cardiovascular diseases (CVD) is intricate. On the one hand, the Klotho protein is closely linked to CKD,^[9]^ and on the other hand, some association of Klotho with CVD was found in CKD patients. Studies have revealed that FGF23 and Klotho have a common effect in CKD patients, regulating mineral metabolism and participating in the occurrence and death of CVD.^[13]^ Six et al^[14]^ found that elevated Klotho and FGF23 levels could induce the contraction of aortic rings and the production of oxidative stress substances in vascular smooth muscle cells. At the same time, Klotho protein can partially reverse the vasoconstriction caused by FGF23, promote aortic relaxation (both may be achieved by increasing NO secretion), and increase the production of NO in human vein endothelial cells. Therefore, Klotho can reduce vasoconstriction and protect blood vessels to some extent.

Previously, evaluating the concentration of serum low-density lipoprotein cholesterol (LDL-C) was a critical factor in managing the risk of atherosclerotic cardiovascular disease (ASCVD).^[15,16]^ However, recent evidence suggests that while lipid-lowering therapy targeting serum LDL-C reduces the risk of ASCVD in the population, some individuals with normal or low serum LDL-C concentrations still experience ASCVD-related events, and some individuals experience atherosclerosis progression.^[17]^ This indicates that it is not the best strategy for all patients to manage ASCVD only by focusing on serum LDL-C concentration and that attention should also be paid to the impact of apolipoprotein B (ApoB). ApoB is a granular apolipoprotein that can be found in a variety of lipoproteins. Each lipoprotein contains 1 molecule of apoB, which is surrounded by a single layer of phospholipids.^[18]^ ApoB provides structural stability to the lipoprotein particles and remains unchanged throughout the metabolic process. The core of the lipoprotein particles consists of varying amounts of triglycerides and cholesterol.^[19]^ ApoB acts as a carrier and is present in different types of lipoproteins, including LDL, intermediate-density lipoprotein, very low-density lipoprotein (VLDL), chyle granules, and LP(a). ApoB100 is mainly found in LDL, while the rest is found in VLDL, intermediate-density lipoprotein, and LP(a), and apoB48 can be found in chyle granules. As each lipoprotein particle contains 1 molecule of apoB, the plasma apoB is equal to the total number of apoB48, apoB100, and LP(a) particles.^[20]^ Since apoB48 is rare, the clinical measurement of apoB equals the concentration of apoB100. The total amount of apoB is the sum of VLDL, LDL, and LP(a) particles, with LDL accounting for the highest amount. This makes apoB highly correlated with LDL. As elevated apoB may predict CVD risk in individuals with normal or low LDL-C levels, exploring the relationship between apoB and Klotho is beneficial for screening potential CVD patients in this population. Currently, some guidelines suggest using apoB as a secondary intervention target of blood lipid management to reduce the residual risk of ASCVD in patients.^[21,22]^ Therefore, apoB plays an essential role in the diagnosis and treatment of CVD.

Based on the contextual information, it is plausible to suggest a potential correlation between serum Klotho and apoB, which requires further exploration. Nevertheless, the existing research on the relationship between serum Klotho and apoB is limited. Therefore, this study aims to investigate the association between serum Klotho and apoB, using data collected from the National Health and Nutrition Examination Survey (NHANES) conducted between 2007 and 2016.

## 2. Methods

### 2.1. Study design and population

The NHANES is a comprehensive health and nutrition survey conducted by the National Center for Health Statistics in the United States. Since its inception in the early 1960s, NHANES has aimed to assess the health and nutritional status of individuals across the United States. This survey utilizes family interviews and physical examinations to gather detailed biological, social, psychological, behavioral, and demographic information at no cost to participants. In this cross-sectional study, we utilized NHANES data from 2007 to 2016 to investigate the relationship between apoB and serum Klotho in the general population.

### 2.2. Sample selection

**Figure 1.**
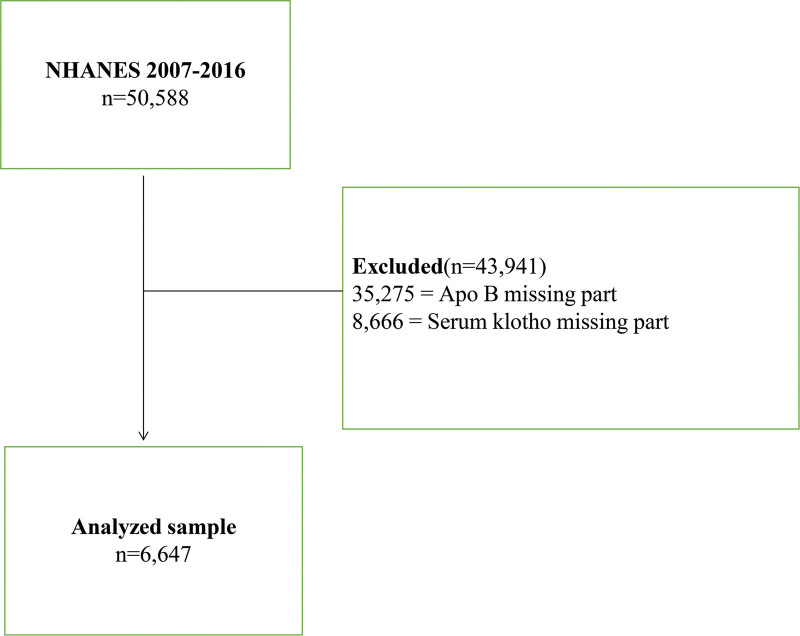
Flow chart of the screening process for the selection of eligible participants.

We analyzed data from the NHANES database, which included 50,588 participants from the years 2007 to 2016. The analysis included demographic data such as age, gender, race, ratio of family income to poverty, and education level. Additionally, we looked at CVD risk factors, including hypertension, high cholesterol levels, diabetes, body mass index (BMI), drinking, and smoking variables. We also examined cancer as part of our analysis. Participants who were missing serum Klotho and apoB were excluded, leaving us with 6647 participants with complete data (as depicted in Fig. 1). All NHANES research participants from 2007 to 2016 provided informed consent and obtained approval from the Research Ethics Review Committee of the National Health Statistics Center.

### 2.3. Covariates

The demographic variables included age, Gender (male or female), Race (Mexican American, other Hispanic, non-Hispanic White, non-Hispanic Black, or Other Race), Ratio of family income to poverty, and Education level (High School Grad and Less Than, or above). Age is a continuous variable in the demographic data. Then, age was categorized into the following 3 groups: <=40 years old, 41 to 60 years old, and >60 years old. Less Than 9th Grade, 9th to 11th Grade (Includes 12th grade with no diploma), and High School Grad/GED or Equivalent were classified as High School Grad and Less Than group. Some College or AA degrees and College graduates or above were classified as high school grad above group. Therefore, education level was classified as High School Grad and Less Than and above.

The questionnaire variables included Hypertension (No or Yes), High cholesterol level (No or Yes), Diabetes (No or Yes), Drinking (No or Yes), Smoking (No or Yes), and Cancer (No or Yes). The group with a drinking frequency of 0 was classified as the non-drinking group, and the group of people with a drinking frequency >0 was classified as the drinking group. Daily and occasional smokers were classified as smoker and never smokers as nonsmokers. Therefore, Smoking status was classified as smokers and never smokers.

The Examination variable included BMI. BMI, as a continuous variable, was categorized into the following 3 groups: underweight (<18.5 kg/m^2^), normal obese (≥25 kg/m^2^), and middle weight (18.5–24.9 kg/m^2^).

### 2.4. Data collection

Trained interviewers conducted a questionnaire survey to collect data on Gender, Age, Race, Education level, Ratio of family income to poverty, Hypertension, High cholesterol level, BMI, Diabetes, Drinking, Smoking, and Cancer. Participants also underwent physical examinations and blood samples were collected to measure apoB (g/L) and Klotho (pg/mL) levels. Details of the NHANES laboratory methodology for Klotho determination are available at: https://wwwn.cdc.gov/Nchs/Nhanes/2007-2008/SSKL_E.htm. Details of the NHANES laboratory methodology for apoB determination are available at: https://wwwn.cdc.gov/Nchs/Nhanes/2007-2008/APOB_E.htm. Covariates can be found at the following URL: www.cdc.gov/nchs/nhanes/.

### 2.5. Data analysis

To study the associations of participant characteristics, we utilized weighted linear regression and the Chi-square test where appropriate. Firstly, we conducted a weighted univariate analysis to examine the relationship between Klotho and the covariates. Next, we performed weighted multifactor analyses while adjusting for covariates such as Gender, Age, Race, Education level, Ratio of family income to poverty, Hypertension, High cholesterol level, BMI, Diabetes, Drinking, Smoking, and Cancer. Additionally, we conducted smooth curve fitting using apoB (g/L) as the abscissa and Klotho (pg/mL) level as the ordinate to confirm the relationship between apoB and serum Klotho.

All statistical analyses were carried out using Empowerstats (https://www.empowerstats.net/cn/) and R software, and all estimates were weighted using the appropriate NHANES sample weight. We utilized weighted models to account for the oversampling of minorities, as suggested by the Centers for Disease Control and Prevention, to ensure a final unbiased and accurate estimate of effects for the population. Findings were deemed statistically significant at a *P* value of <.05.

## 3. Results

The table shows 14 study variables, which are composed of Serum Klotho, ApoB, Gender, Age, Race, Education level, Ratio of family income to poverty, Hypertension, High cholesterol level, BMI, Diabetes, Drinking, Smoking, and Cancer. A total of 6647 participants were selected, with apoB as the independent variable, and they were divided into 3 groups for research (T1 group N = 2147; T2 group = 2195; T3 group = 2305) (Table 1).

**Table 1:** Characteristics of participants, weighted (N = 6647).

ApoB (g/L) tertile	T1 <0.8 g/L	T2 ≥0.8 g/L to <1.0 g/L	T3 ≥1.0 g/L	*P* value
N	2147	2195	2305	
Serum klotho (pg/mL)	867.2 (843.4, 891.0)	856.4 (837.6, 875.1)	823.1 (804.9, 841.3)	.004
ApoB (g/L)	0.7 (0.7, 0.7)	0.9 (0.9, 0.9)	1.2 (1.2, 1.2)	<.001
Ratio of family income to poverty	3.3 (3.2, 3.4)	3.2 (3.1, 3.4)	3.1 (3.0, 3.3)	.108
Gender				.301
Male	46.0 (43.3, 48.7)	47.4 (44.9, 49.9)	49.0 (46.7, 51.3)	
Female	54.0 (51.3, 56.7)	52.6 (50.1, 55.1)	51.0 (48.7, 53.3)	
Age (yr)				<.001
<=40	3.6 (2.7, 4.6)	2.5 (1.9, 3.4)	2.8 (2.1, 3.6)	
>40, <=60	56.7 (53.5, 59.9)	61.0 (58.1, 63.8)	67.8 (65.2, 70.4)	
>60	39.7 (36.7, 42.8)	36.4 (33.7, 39.3)	29.4 (27.0, 32.0)	
Race				<.001
Mexican American	5.2 (4.0, 6.8)	6.6 (5.1, 8.5)	7.5 (6.0, 9.3)	
Other Hispanic	3.6 (2.7, 4.8)	4.7 (3.7, 6.0)	6.3 (4.8, 8.3)	
Non-Hispanic White	74.5 (71.1, 77.6)	73.5 (69.9, 76.9)	73.1 (69.2, 76.6)	
Non-Hispanic Black	10.1 (8.4, 12.1)	8.4 (7.1, 10.0)	7.5 (6.1, 9.3)	
Other Race	6.6 (5.5, 8.0)	6.7 (5.3, 8.5)	5.6 (4.5, 7.1)	
Education level				<.001
High school grad and less than	34.3 (30.5, 38.3)	36.9 (33.6, 40.4)	42.9 (38.9, 47.0)	
Above	65.7 (61.7, 69.5)	63.1 (59.6, 66.4)	57.1 (53.0, 61.1)	
Hypertension				.774
No	56.2 (53.2, 59.1)	57.5 (54.3, 60.6)	57.4 (54.9, 59.9)	
Yes	43.8 (40.9, 46.8)	42.5 (39.4, 45.7)	42.6 (40.1, 45.1)	
High cholesterol level				<.001
No	59.3 (56.5, 62.1)	55.6 (52.7, 58.5)	40.4 (37.6, 43.4)	
Yes	40.7 (37.9, 43.5)	44.4 (41.5, 47.3)	59.6 (56.6, 62.4)	
BMI (kg/m^2^)				<.001
<=18.5	1.6 (1.0, 2.6)	1.0 (0.5, 1.9)	0.2 (0.1, 0.4)	
>18.5, <=25	24.0 (21.2, 27.1)	18.0 (15.6, 20.8)	15.2 (13.0, 17.7)	
>25	74.3 (71.1, 77.3)	81.0 (78.1, 83.6)	84.6 (82.1, 86.8)	
Diabetes				<.001
No	82.9 (80.9, 84.7)	87.5 (85.4, 89.4)	89.5 (87.7, 91.1)	
Yes	17.1 (15.3, 19.1)	12.5 (10.6, 14.6)	10.5 (8.9, 12.3)	
Drinking				.031
No	20.8 (18.3, 23.6)	18.1 (15.7, 20.8)	22.6 (19.9, 25.6)	
Yes	79.2 (76.4, 81.7)	81.9 (79.2, 84.3)	77.4 (74.4, 80.1)	
Smoking				.004
No	66.2 (61.3, 70.7)	65.8 (61.2, 70.2)	58.0 (53.7, 62.2)	
Yes	33.8 (29.3, 38.7)	34.2 (29.8, 38.8)	42.0 (37.8, 46.3)	
Cancer				.917
No	87.4 (85.6, 89.1)	86.9 (84.9, 88.7)	87.0 (85.0, 88.9)	
Yes	12.6 (10.9, 14.4)	13.1 (11.3, 15.1)	13.0 (11.1, 15.0)	

Data in the Table 1:

For continuous variables: survey-weighted mean (95% CI), *P* value was by survey-weighted linear regression (svyglm).

For categorical variables: survey-weighted percentage (95% CI), *P* value was by survey-weighted Chi-square test (svytable).

ApoB = apolipoprotein B.

In the single-factor regression analysis, 13 variables of the study are displayed by serum Klotho. As can be seen from Table 2, in addition to apoB and Klotho having a certain correlation, gender, age, race, and high cholesterol levels are also related to Klotho.

**Table 2:** Univariate analysis for serum klotho, weighted.

Covariate	Mean/percentage (95% CI)	Β (95%CI)	*P* value
ApoB (g/L)	1.0 (0.9, 1.0)	−71.7 (−120.8, −22.6)	.005
ApoB (g/L) Tertile			
Low	32.1 (30.2, 34.0)	Ref	
Middle	33.4 (31.9, 35.0)	−10.9 (−38.8, 17.1)	.448
High	34.5 (32.8, 36.2)	−44.1 (−72.6, −15.6)	.003
Ratio of family income to poverty	3.2 (3.1, 3.3)	−1.0 (−8.2, 6.2)	.794
Ratio of family income to poverty tertile			
Low	19.6 (17.6, 21.8)	Ref	
Middle	31.1 (29.0, 33.2)	−5.0 (−32.5, 22.3)	.717
High	49.3 (46.1, 52.5)	−5.5 (−33.6, 22.6)	.702
Gender			
Male	47.5 (46.3, 48.7)	Ref	
Female	52.5 (51.3, 53.7)	45.6 (27.7, 63.5)	<.001
Age (yr)	56.4 (56.0, 56.8)	−1.5 (−2.2, −0.7)	<.001
Age (yr) tertile			
<=40	2.9 (2.5, 3.4)	Ref	
>40, <=60	62.0 (60.2, 63.8)	−23.2 (82.0, 35.6)	.442
>60	35.1 (33.4, 36.8)	−62.0 (-−116.4, −7.7)	.028
Race			
Mexican American	6.5 (5.1, 8.1)	Ref	
Other Hispanic	4.9 (3.8, 6.3)	12.3 (−23.8, 48.3)	.507
Non-Hispanic White	73.7 (70.4, 76.7)	−32.1 (−57.4, −6.8)	.015
Non-Hispanic Black	8.7 (7.3, 10.2)	53.4 (18.2, 88.6)	.004
Other Race	6.3 (5.4, 7.4)	10.8 (−25.6, 47.2)	.562
Education level			
High school grad and less than	38.1 (35.3, 41.0)	Ref	
Above	61.9 (59.0, 64.7)	9.1 (−9.9, 28.2)	.350
Hypertension			
No	57.0 (55.3, 58.8)	Ref	
Yes	43.0 (41.2, 44.7)	−12.5 (−34.8, 9.7)	.274
High cholesterol level			
No	51.6 (49.9, 53.3)	Ref	
Yes	48.4 (46.7, 50.1)	−29.4 (−51.2, −7.6)	.010
BMI (kg/m^2^)	57.4 (54.1, 60.7)	−0.1 (−0.3, 0.1)	.385
BMI (kg/m^2^) Tertile			
<=18.5	0.9 (0.7, 1.3)	Ref	
>18.5, <=25	19.0 (17.3, 20.8)	23.3 (−82.5, 129.1)	.667
>25	80.1 (78.2, 81.9)	−5.4 (−101.4, 90.4)	.911
Diabetes			
No	86.7 (85.3, 88.0)	Ref	
Yes	13.3 (12.0, 14.7)	−7.6 (32.0, 16.7)	.541
Drinking			
No	20.6 (18.8, 22.4)	Ref	
Yes	79.4 (77.6, 81.2)	−4.0 (−28.3, 20.4)	.750
Smoking			
No	63.1 (59.9, 66.1)	Ref	
Yes	36.9 (33.9, 40.1)	−14.9 (−38.4, 8.5)	.216
Cancer			
No	87.1 (85.9, 88.2)	Ref	
Yes	12.9 (11.8, 14.1)	−25.2 (−53.4, 3.0)	.084

ApoB = apolipoprotein B, CI = confidence interval, Ref = reference.

The result shown in Table 3 for the model revealed a negative association between apoB concentration and the concentration of klotho in the serum in the non-adjustment model (β = −71.7; 95% CI: −120.8, −22.6; *P* = .005), adjusted model (β = −91.8; 95% CI: −151.3, −32.2; *P* = .004). The conversion of the apoB concentration from a continuous variable to a categorical variable (tertile: T1 <0.8 g/L; T2: 0.8–1.0 g/L; T3: ≥1.0 g/L) revealed that the level of Klotho in the serum of the participants in the highest tertile (≥1.0 g/L) was −44.8 pg/mL (95% CI: −86.3, −3.2; *P* = .040) lower than that in the lowest tertile (<0.8 g/L).

**Table 3:** Relationship between ApoB and serum klotho, weighted.

Outcome	Crude model		Adjusted model	
	Β or RR (95%CI)	*P* value	Β or RR (95%CI)	*P* value
ApoB (g/L)	−71.7 (−120.8, −22.6)	.005	−91.8 (−151.3, −32.2)	.004
ApoB (g/L) Tertile				
T1 (<0.8 g/L)	Ref		Ref	
T2 (≥0.8 g/L to <1.0 g/L)	−10.9 (−38.8, 17.1)	.448	−1.5 (−50.2, 47.1)	.951
T3 (≥1.0 g/L)	−44.1 (−72.6, −15.6)	<.001	−44.8 (−86.3, −3.2)	.040

ApoB = apolipoprotein B, CI = confidence interval, Ref = reference.

Data in the table: β or RR (95%CI); *P* value.

Result variable: Serum Klotho (pg/mL).

Exposure variable: ApoB (g/L); ApoB (g/L) Tertile.

The adjusted model adjusts for Gender (Male, Female); Age (years) (<=40, >40, <=60, >60); Race (Mexican American, Other Hispanic, Non-Hispanic White, Non-Hispanic Black, Other Race); Ratio of family income to poverty; Hypertension (No, Yes); High cholesterol level (No, Yes); BMI (kg/m^2^) (<=18.5, >18.5, <=25, >25); Diabetes (No, Yes); Drinking (No, Yes); Smoking (No, Yes); Cancer (No, Yes).

**Figure 2.**
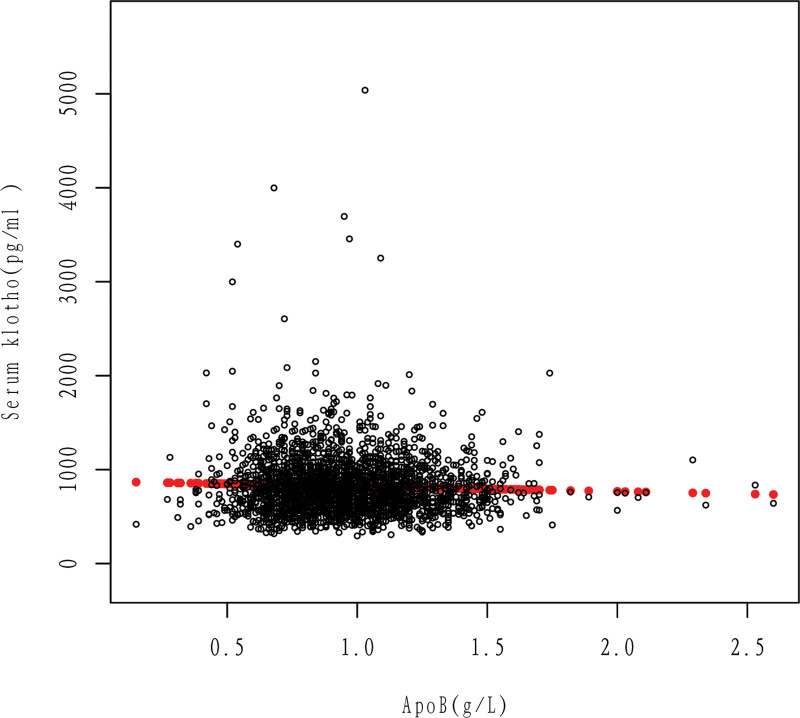
Association between apoB and serum klotho. Cross-combination modeling of each apoB and Klotho. A Black dot represents a sample. ApoB = apolipoprotein B.

**Figure 3.**
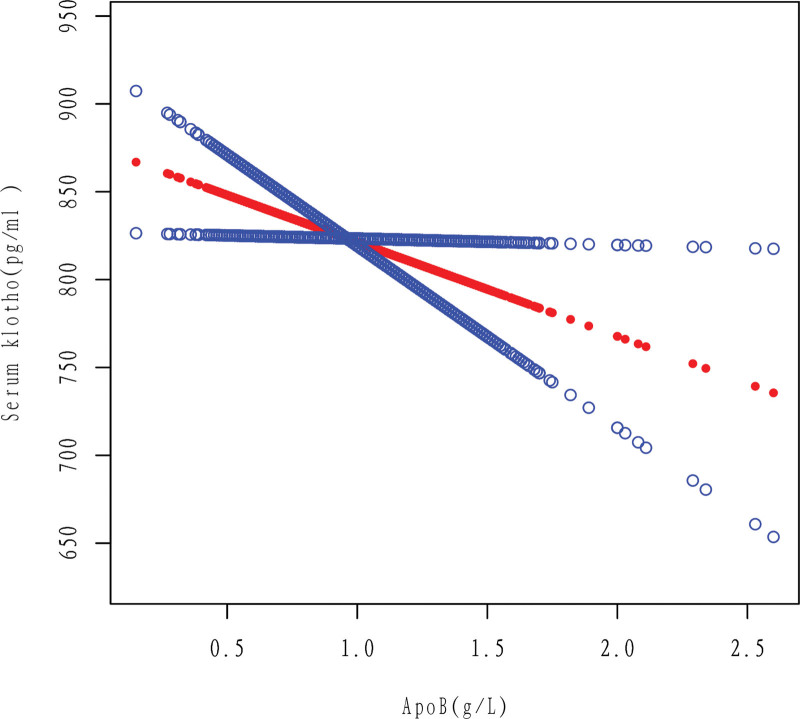
Association between apoB and serum klotho. A linear association between apoB and serum klotho was found in a generalized additive model (GAM). The Solid red line represents the smooth curve fit between variables. Blue bands represent the 95% of confidence interval from the fit. The results were obtained after adjusting for Gender, Age, Race, Education level, Ratio of family income to poverty, Hypertension, High cholesterol level, BMI, Diabetes, Drinking, Smoking, and Cancer. It can be seen that after adjusting the relevant variables, apoB was linearly correlated with Klotho, and Klotho decreased with the increase of apoB. ApoB = apolipoprotein B.

It can be seen that after adjusting the relevant variables, apoB was linearly correlated with Klotho, and Klotho decreased with the increase of apoB (Figs. 2 and 3).

**Figure 4.**
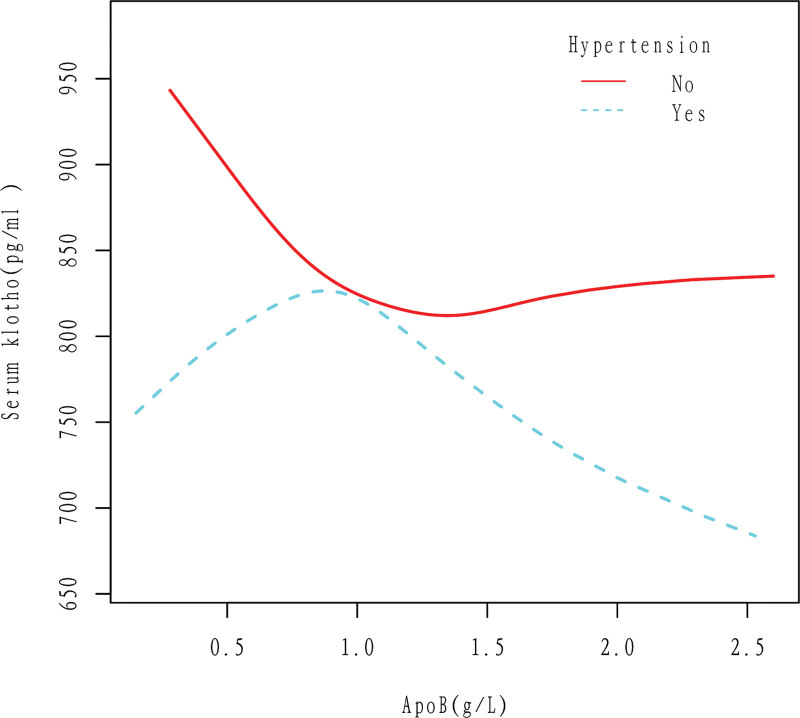
Association between apoB and serum Klotho in Hypertension (No, Yes) group. In the hypertensive population, Klotho increases first and then decreases with the increase of apoB. In the non-hypertensive population, Klotho showed a downward trend with increasing apoB, and then the downward trend tended to slow down. The results were obtained after adjusting for Gender, Age, Race, Education level, Ratio of family income to poverty, High cholesterol level, BMI, Diabetes, Drinking, Smoking, and Cancer. ApoB = apolipoprotein B.

In the hypertensive population, Klotho increases first and then decreases with the increase of apoB. In the non-hypertensive population, Klotho showed a downward trend with increasing apoB, and then the downward trend tended to slow down. The results were obtained after adjusting for Gender, Age, Race, Education level, Ratio of family income to poverty, High cholesterol level, BMI, Diabetes, Drinking, Smoking, and Cancer (Fig. 4).

**Figure 5.**
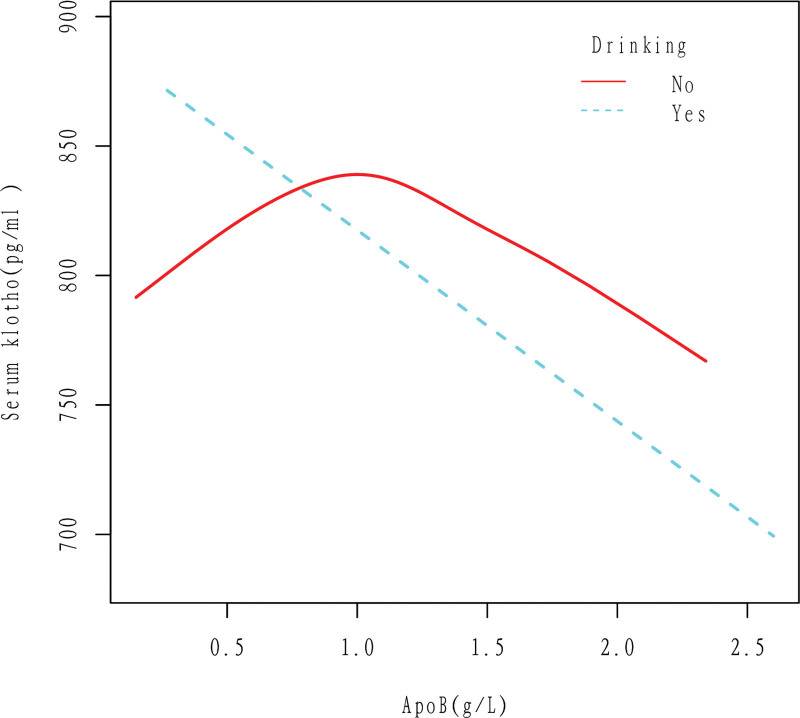
Association between apoB and serum Klotho in Drinking (No, Yes) group. In alcohol drinkers, apoB was linearly correlated with Klotho, and Klotho decreased with the increase of apoB. In the non-alcohol drinkers, Klotho increases first and then decreases with the increase of apoB. The results were obtained after adjusting for Gender, Age, Race, Education level, Ratio of family income to poverty, Hypertension, High cholesterol level, BMI, Diabetes, Smoking, and Cancer. ApoB = apolipoprotein B.

In alcohol drinkers, apoB was linearly correlated with Klotho, and Klotho decreased with the increase of apoB. In the non-alcohol drinkers, Klotho increases first and then decreases with the increase of apoB. The results were obtained after adjusting for Gender, Age, Race, Education level, Ratio of family income to poverty, Hypertension, High cholesterol level, BMI, Diabetes, Smoking, and Cancer (Fig. 5).

**Figure 6.**
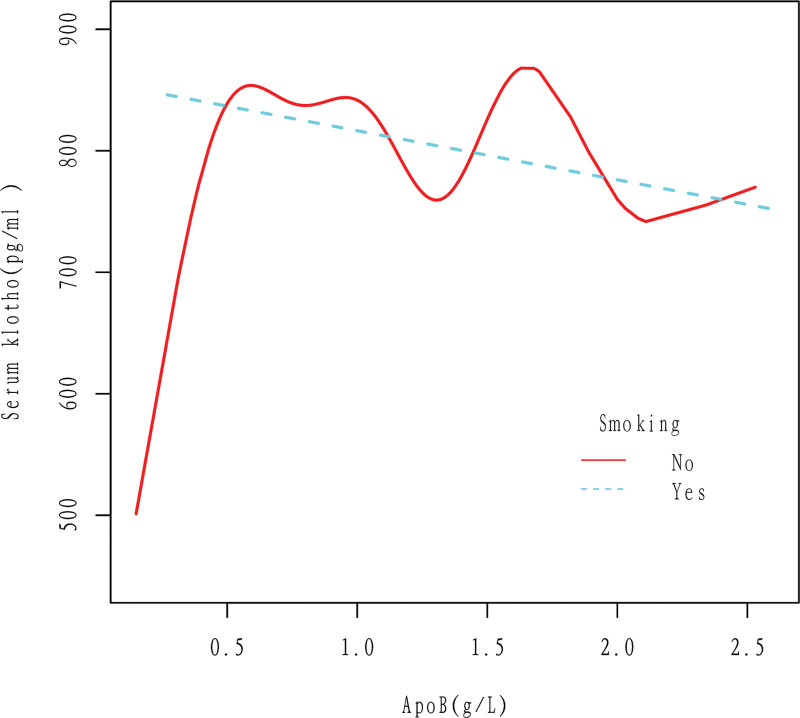
Association between apoB and serum Klotho in Smoking (No, Yes) group. In the smoker population, apoB was linearly correlated with Klotho, and Klotho decreased with the increase of apoB. In the nonsmoker population, The relationship between apoB and Klotho loses its linear correlation. The results were obtained after adjusting for Gender, Age, Race, Education level, Ratio of family income to poverty, Hypertension, High cholesterol level, BMI, Diabetes, Drinking, and Cancer. ApoB = apolipoprotein B.

In the smoker population, apoB was linearly correlated with Klotho, and Klotho decreased with the increase of apoB. In the nonsmoker population, The relationship between apoB and Klotho loses its linear correlation. The results were obtained after adjusting for Gender, Age, Race, Education level, Ratio of family income to poverty, Hypertension, High cholesterol level, BMI, Diabetes, Drinking, and Cancer (Fig. 6).

**Figure 7.**
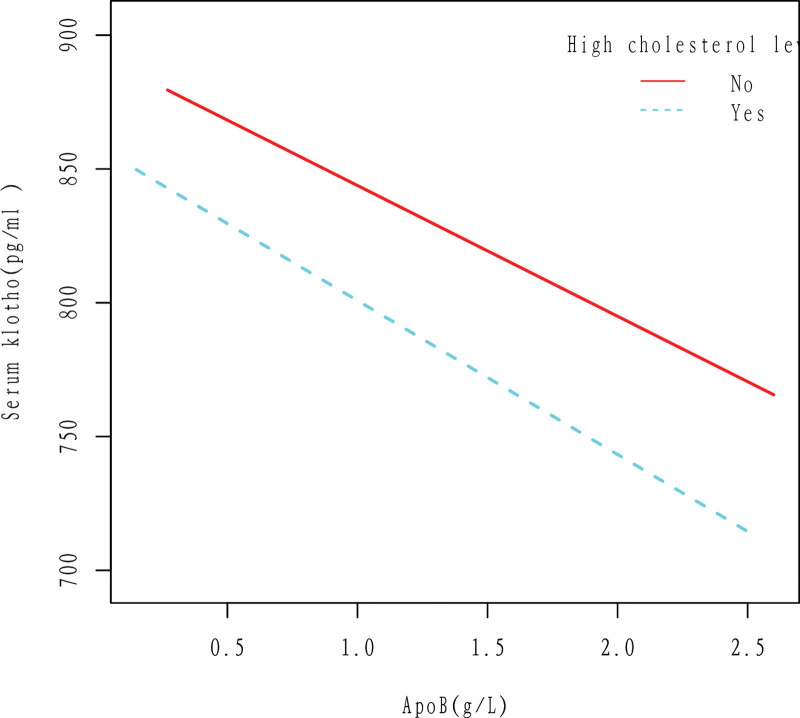
Association between apoB and serum Klotho in High cholesterol level (No, Yes) group. ApoB was linearly correlated with Klotho, and Klotho decreased with the increase of apoB in both high cholesterol level and non-high cholesterol level populations. The results were obtained after adjusting for Gender, Age, Race, Education level, Ratio of family income to poverty, Hypertension, BMI, Diabetes, Drinking, Smoking, and Cancer. ApoB = apolipoprotein B.

ApoB was linearly correlated with Klotho, and Klotho decreased with the increase of apoB in both high cholesterol level and non-high cholesterol level populations. The results were obtained after adjusting for Gender, Age, Race, Education level, Ratio of family income to poverty, Hypertension, BMI, Diabetes, Drinking, Smoking, and Cancer (Fig. 7).

**Figure 8.**
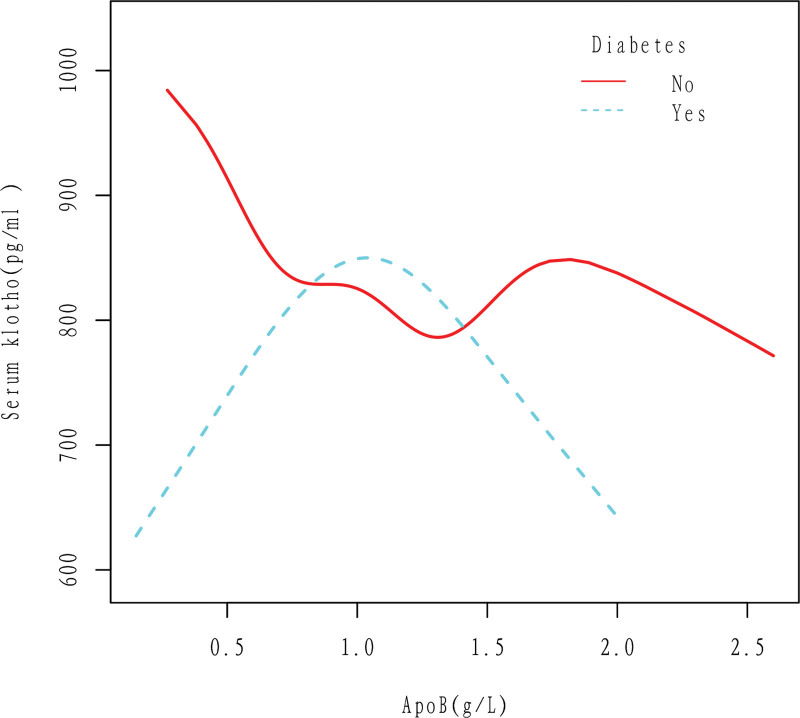
Association between apoB and serum Klotho in Diabetes (No, Yes) group. In diabetic patients, Klotho first increased and then decreased with the increase of apoB. In nondiabetic patients, the curve relationship between apoB and Klotho is not fixed, but overall Klotho decreases with the increase of apoB. The results were obtained after adjusting for Gender, Age, Race, Education level, Ratio of family income to poverty, Hypertension, BMI, High cholesterol level, Drinking, Smoking, and Cancer. ApoB = apolipoprotein B.

In diabetic patients, Klotho first increased and then decreased with the increase of apoB. In nondiabetic patients, the curve relationship between apoB and Klotho is not fixed, but overall Klotho decreases with the increase of apoB. The results were obtained after adjusting for Gender, Age, Race, Education level, Ratio of family income to poverty, Hypertension, BMI, High cholesterol level, Drinking, Smoking, and Cancer (Fig. 8).

## 4. Discussion

According to this study, there is a negative linear correlation between apoB and Klotho in the general population, as shown in Figures 2 and 3. Specifically, an increase in apoB is associated with a decrease in Klotho levels. This relationship holds true even when several cardiovascular risk factors are present, such as drinking, smoking, and high cholesterol level (Figs. 5–7). However, the relationship between apoB and Klotho in hypertensive and diabetic populations follows a different pattern (Figs. 4 and 8). In these populations, Klotho initially increases with an increase in apoB before decreasing, as seen in Figures 4 and 8. Importantly, the correlation between apoB and Klotho remains significant even after adjusting for potential confounders. This study is supported by 2 key theoretical concepts: the high correlation between apoB and LDL-C, and the role of apoB as a potential risk factor for CVD.^[20]^ As such, elevated levels of apoB may serve as an important predictor of CVD risk, particularly in individuals with normal or low LDL-C levels. Therefore, further exploration of the relationship between apoB and Klotho may be a useful tool for identifying potential CVD patients within this population.

To the best of our knowledge, this study is the first to investigate the association between apoB concentration and serum Klotho concentration in the general population of the United States. Our findings suggest that specific CVD risk factors may be linked to lower Klotho levels, and controlling modifiable factors may help prevent or alleviate the decline of Klotho. The expression level of Klotho protein is also related to aging, and Kuro-o found that mice lacking Klotho suffer from premature aging syndrome.^[23]^ Previous evidence has shown that Klotho deficiency and CKD cause aging, CVD, and bone disease.^[24]^ Furthermore, there is a strong association between aging and the incidence of several age-related chronic diseases, such as diabetes and inflammatory bowel disease.^[25,26]^ Thus, an unexpected discovery was made that apoB may be related to aging.

Several studies are currently investigating the potential association between the Klotho protein and dyslipidemia. Previous research has demonstrated that decreased serum Klotho levels and Klotho gene expression in the coronary artery wall are linked to the severity of coronary heart disease.^[27]^ Animal studies have also indicated that Klotho protein could potentially promote adipocyte differentiation and play an anti-atherosclerotic role, decreasing the risk of CVD.^[28]^ Recent studies have found a significant correlation between Klotho and blood lipids such as total cholesterol and triglyceride.^[29]^ However, to date, there has been no investigation into the relationship between apoB and Klotho. In this study, we examined the correlation between apoB and Klotho while considering a range of potential confounding factors, including gender, age, race, education level, ratio of family income to poverty, hypertension, high cholesterol level, BMI, diabetes, drinking, smoking, and cancer. We utilized a linear regression model and smooth curve fitting to assess the association between apoB concentration and serum Klotho concentration and discovered a negative correlation between the 2.

This study stands out for being the pioneer in using public data from NHANES to explore the correlation between serum Klotho and the potential risk factor for CVD, apoB. Despite its strengths, it is important to acknowledge that this study has some limitations. Firstly, due to the cross-sectional nature of the NHANES database, we cannot infer any causal relationship between apoB and Klotho in humans. Secondly, there is a possibility of unknown confounding variables that could affect serum Klotho and apoB levels and were not considered in the study.

## 5. Conclusions

The Klotho protein is believed to play a critical role in regulating the onset and progression of CVD and other systemic illnesses. Investigating the function of Klotho and its impact on blood biochemical markers can be highly valuable in uncovering its role in CVD risk factors, thereby guiding clinical and prospective scientific research. Gene therapy targeting the Klotho gene may also hold promise in delaying aging and enhancing the quality of life of patients. With apoB high correlation with LDL-C and its potential as a CVD risk factor, identifying individuals at risk of CVD among the population with normal or low LDL-C levels by exploring the relationship between apoB and Klotho could be advantageous. A low serum Klotho level is associated with apoB, indicating that the soluble Klotho level may serve as a potential therapeutic marker for CVD prevention. However, more research is needed to clarify the Klotho gene precise control mechanism and the relationship between the specific transfer signal of the Klotho gene protein and cardiovascular risk factors.

## Acknowledgments

We would like to thank all the participants for their contributions. In particular, we would like to recognize the contributions of Tao Tao and Guixiao Huang. Thank you, Mr. Tao Tao, for your kind guidance, and Mr. Guixiao Huang for providing a precious platform.

## Author contributions

**Conceptualization:** Guixiao Huang, Tao Tao, Zhiyi Chen.

Data curation: Zhiyi Chen.

Investigation: Zhiyi Chen, Xin Tong, Qinhe Li, Guanyu Su.

Supervision: Tao Tao, Guixiao Huang.

Writing – original draft: Zhiyi Chen.

Writing – review & editing: Zhiyi Chen, Tao Tao.
